# P-1766. Evaluating the Utility of Serum Procalcitonin for Treatment Duration of Bloodstream Infections – A Sub-study of the BALANCE Trial

**DOI:** 10.1093/ofid/ofaf695.1936

**Published:** 2026-01-11

**Authors:** Rayoun Ramendra, Julie Wright, Ruxandra Pinto, Kevin Kain, Robert Fowler, Nick Daneman

**Affiliations:** University of Toronto, Toronto, Ontario, Canada; University Health Network, Toronto, Ontario, Canada; University of Toronto, Toronto, Ontario, Canada; University Health Network, Toronto, Ontario, Canada; Sunnybrook Health Sciences Centre, ON, Canada; Sunnybrook Health Sciences Centre, University of Toronto, Toronto, Ontario, Canada

## Abstract

**Background:**

The BALANCE multicenter randomized clinical trial found that 7 days was non-inferior to 14 days of antibiotic treatment for bloodstream infections. Procalcitonin (PCT) is a protein produced by leukocytes in response to bacterial, but not viral, infections. Accordingly, it has been hypothesized to be a specific biomarker of bacterial infection. Several studies have investigated the clinical utility of measuring serum PCT levels to predict infection severity and personalize treatment duration but have produced conflicting results. In this planned sub-study of BALANCE, we evaluated whether day 7 PCT serum level was associated with mortality in patients treated with either 7 days or 14 days of antibiotics.

**Methods:**

Day 7 serum PCT was measured in 125 patients enrolled in the BALANCE trial. Based on previous studies, a cut-off of 250 pg/mL was used to determine low and high levels of PCT. Baseline patient demographics, infection characteristics, and 90-day mortality were compared between patients with low and high PCT. Among individuals with high PCT (predicted greater infection severity) treated with 7 or 14 days of antibiotic treatment, we calculated the 90-day mortality absolute risk difference (ARD) and 95% confidence interval (CI).

**Results:**

A total of 65 (52%) patients had low PCT and 60 (48%) patients had high PCT. Individuals with high PCT were older, had more comorbidities, and were more likely to have community-acquired bacteremia. By 90 days, 4/65 (6.1%) patients with low PCT and 13/60 (22%) patients with high PCT had died (ARD 0.15; 95% CI, 0.04 to 0.28). Among patients with high PCT, 3/27 (11.1%) of patients with 7-day treatment and 10/33 (30.3%) of patients with 14-day treatment had died by 90 days (ARD -0.19; 95% CI, -0.39 to 0.005).

**Conclusion:**

In this sub-study of the BALANCE trial, high serum day 7 PCT level was associated with increased 90-day mortality. However, among patients with high PCT, longer duration of antibiotic treatment was not associated with improved outcome. Seven days of antibiotics may be sufficient for patients with bloodstream infections independent of serum PCT level.

**Disclosures:**

All Authors: No reported disclosures

